# Comparative physiological and root proteome analyses of two sorghum varieties responding to water limitation

**DOI:** 10.1038/s41598-020-68735-3

**Published:** 2020-07-16

**Authors:** Tatenda Goche, Nemera G. Shargie, Ian Cummins, Adrian P. Brown, Stephen Chivasa, Rudo Ngara

**Affiliations:** 1 Department of Plant Sciences, University of the Free State, Qwaqwa Campus, P. Bag X13, Phuthaditjhaba, South Africa; 2 Agricultural Research Council-Grain Crops Institute, P. Bag X1251, Potchefstroom, 2520 South Africa; 3 Department of Biosciences, Durham University, South Road, Durham, DH1 3LE UK

**Keywords:** Abiotic, Drought

## Abstract

When exposed to drought stress many plants reprogram their gene expression to activate adaptive biochemical and physiological responses for survival. However, most of the well-studied adaptive responses are common between drought-sensitive and drought-tolerant species, making it difficult to identify the key mechanisms underpinning successful drought tolerance in crops. We developed a sorghum experimental system that compares between drought-sensitive (ICSB338) and enhanced drought-tolerant (SA1441) varieties. We show that sorghum activates a swift and robust stomatal shutdown to preserve leaf water content when water stress has been sensed. Water uptake is enhanced via increasing root cell water potential through the rapid biosynthesis of predominantly glycine betaine and an increased root-to-shoot ratio to explore more soil volume for water. In addition to stomatal responses, there is a prompt accumulation of proline in leaves and effective protection of chlorophyll during periods of water limitation. Root and stomatal functions rapidly recover from water limitation (within 24 h of re-watering) in the drought-tolerant variety, but recovery is impaired in the drought-sensitive sorghum variety. Analysis of the root proteome revealed complex protein networks that possibly underpin sorghum responses to water limitation. Common and unique protein changes between the two sorghum varieties provide new targets for future use in investigating sorghum drought tolerance.

## Introduction

Drought stress is a major barrier to agricultural productivity^1^, primarily causing damaging water deficits in plant cells. Drought-induced osmotic stress triggers loss of cell turgor, which affects the rate of cell expansion and overall cell size, ultimately restricting plant growth and development^2^. In sub-Saharan Africa, drought is often associated with very high temperatures, the combination of which severely limits crop productivity^1^, particularly in rain-fed agricultural systems. Several climatic models predict increases in surface temperatures and drought episodes by the year 2050^3^, further threatening global food security^4^. Therefore, it is important to understand plant adaptive responses to drought in order to improve breeding for drought tolerant crops^5^.

Plants respond to the effects of drought stress through a complex network of mechanisms aimed at sustaining cellular metabolism under the prevailing conditions. The mechanisms of drought avoidance, escape and tolerance in a variety of plant species have been extensively reviewed^2,6–8^. While drought escape is important in desert ephemerals that rapidly complete their life cycles before the onset of water deficits^2^, drought avoidance and tolerance are significant response mechanisms in many other plant species. For instance, plants can avoid drought by conserving water through various anatomical features. These include an extensive root system, a thick waxy cuticle, and reduced leaf surface area attained through leaf rolling, folding or shedding. In addition, changes in stomatal number, size and architecture also reduce water loss, while succulence in leaves, stems and roots helps with water storage^2,6,8^.

Drought tolerance involves stress perception, signal transduction pathways and changes in gene expression, which ultimately alter plant physiology and cellular metabolism^9^. When plants sense a decrease in soil water content, they activate the biosynthesis and accumulation of abscisic acid (ABA). ABA in turn activates stomatal closure and the expression of stress-inducible genes. While stomatal closure regulates the rate of transpirational water loss, the activated genes encode for proteins with diverse functions^10–12^. For example, drought-induced proteins such as late embryogenesis abundant proteins, chaperones and detoxification enzymes directly protect cellular components against osmotic stress-induced damage. Other proteins, such as transcription factors, protein kinases and phospholipase C, have gene regulatory and signaling functions^11,12^. Osmotic-stressed plants also accumulate compatible solutes^9,13,14^ that stabilize proteins^15^ and maintain cell turgor and structure^14^. As a result, cellular metabolism is sustained albeit at reduced capacity under water deficits.

However, the effectiveness of the above-mentioned adaptive responses varies between and within plant species, the developmental stages and on the duration and intensity of the prevailing primary and secondary stresses of drought^6,10^. For example, sorghum [*Sorghum bicolor* (L.) Moench] is a naturally drought tolerant and highly productive C_4_ photosynthetic plant^16–19^. It has a wide phenotypic diversity^20,21^ and variation in the degree of drought tolerance exists within its germplasm. For instance, pre- and post-flowering drought tolerant varieties have been identified^16^ and the post-flowering drought tolerant lines are closely associated with the stay-green trait^22^. Furthermore, drought tolerant/resistant sorghum varieties are highly water use efficient (WUE) with an extensive root system for water absorption^23^ and a thick waxy cuticle that retards excessive water loss^17^. Different sorghum varieties also accumulate varying amounts of osmolytes^24–26^ for osmotic adjustment during periods of drought stress.

The genetic basis for sorghum adaptive responses to drought is being studied, and this crop is considered an important source of novel drought genes for crop improvement^21,27^. The sorghum genome has been sequenced^28^ and is part of the growing cereal genomics resource^27,29^, guiding crop breeding strategies. Sorghum physiological^26,30,31^, transcriptomic^32,33^, and proteomic^34–37^ responses to osmotic/drought stress together with computational characterization of candidate genes^38,39^ have been conducted. However, more comparative studies are required in order to fully understand how sorghum survives in hot and dry environments that are unsuitable for most crops. In this study, we analysed the water limitation-induced physiological, growth, biochemical, protein and gene expression changes that occur in two sorghum varieties with contrasting responses to drought. For proteomic analysis of the water limitation response, we focused on root tissue because of the central role it plays in primary stress perception and signaling to the entire plant. Our working hypothesis is that the differential responses to water limitation between the two sorghum varieties are driven by processes within the root systems.

## Results

### ICSB338 and SA1441 seedlings have contrasting drought phenotypes

**Figure 1:**
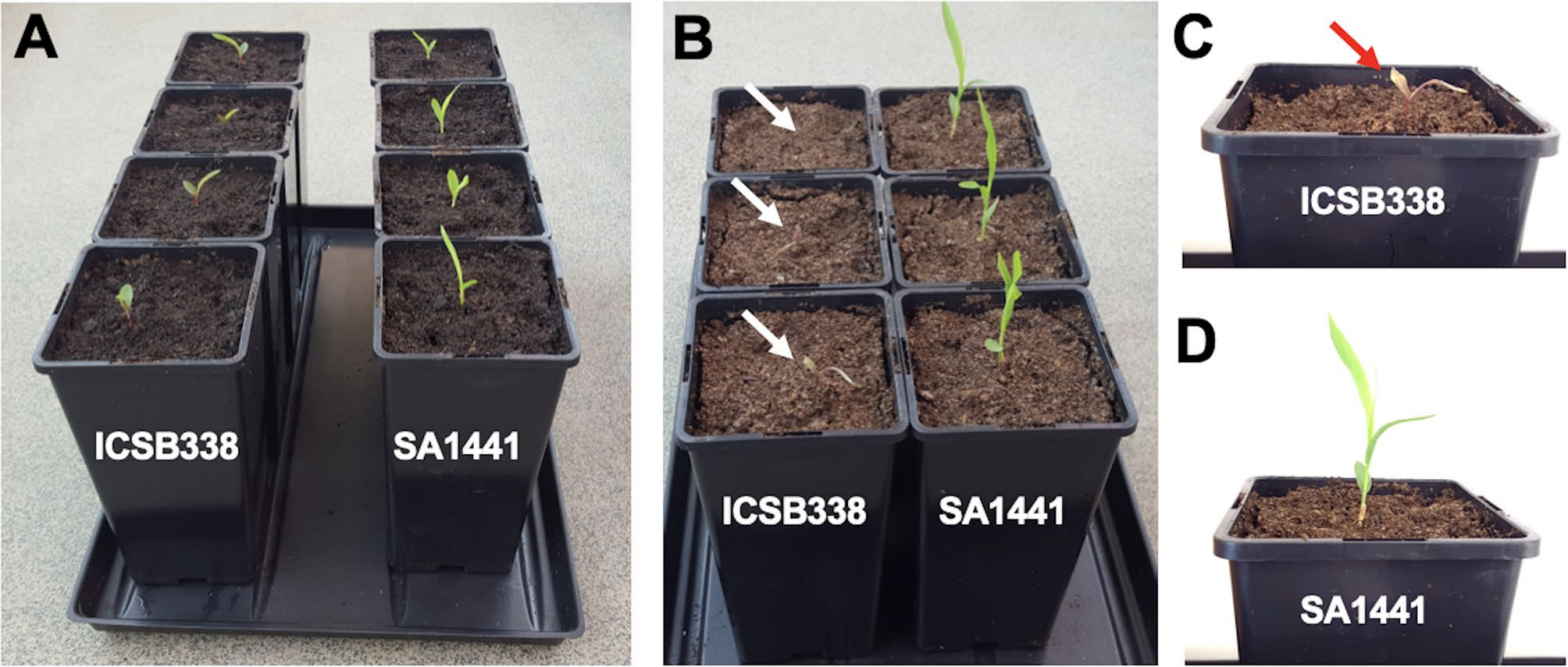
Contrasting drought phenotypes of sorghum seedlings. Four-day old seedlings were transplanted into a column of soil in 2.5 L pots of 22 cm depth and well-watered for establishment. The seedlings were left to grow without any further watering. A photograph was taken a week after transplanting (**A**). Appearance of water-limited plants three weeks after transplanting (**B**). Panels C and D show a close-up of representative plants post-water limitation. White and red arrows show the position of the wilted and dying ICSB338 plants against the soil background.

Although plant breeders originally classified the sorghum varieties used in this study as drought-tolerant (SA1441) and drought-susceptible (ICSB338), there currently is no published data confirming these traits. Therefore, we conducted a water limitation experiment using soil-grown plants in deep pots, to simulate what happens in the field. Four-day-old seedlings germinated in the dark were transplanted into a column of soil in 2.5 L pots of 22 cm depth and the soil fully hydrated. The seedlings were left to establish and grow without any further watering. A photograph taken a week after transplanting reveals that both varieties had successfully established in the soil (Fig. 1A). In this experimental system, air currents in the growth room lead to drying of the pots, from the top to the bottom, mimicking field conditions after rainfall when the top soil layer dries and the water table moves downwards. Because the seedlings were planted in the top soil layer, they are quickly exposed to a soil water deficit and roots that can withstand the stress can continue to grow downwards in pursuit of the receding water column. Starting from 2 weeks after transplanting, ICSB338 plants started to wilt and were completely dead at the 3 weeks point when the experiment was terminated (Fig. 1B). Therefore, ICSB338 plants are sensitive to water limitation and eventually die (Fig. 1C), while drought-tolerant SA1441 plants survive (Fig. 1D). This experiment confirmed the contrasting drought phenotypes of ICSB338 and SA1441 at seedling stage.

### Physiological and growth responses to water limitation

We designed experiments for comparative analysis of water limitation-induced physiological, growth, and biochemical responses and the associated changes in protein and gene expression of two sorghum varieties with contrasting drought phenotypic traits. After germination and initial growth with adequate watering, water limitation was then induced in a soil pot experiment by withholding water supply at the V3 stage (3 fully expanded leaves with the fourth one emerging) for a maximum of 12 days. During the stress treatment period, a range of physiological, growth and biochemical parameters were evaluated in order to establish the extent of water stress and confirm the drought response phenotypes of the two sorghum varieties. Physiological, growth and proteome analyses were conducted following 8 days of water stress, while osmolyte and gene expression analyses were performed in a time-course experiment over 12 days of water limitation. To assess the ability to recover from the stress, some of the plants were re-watered for 24 h following 8 days of limited water supply.

**Figure 2:**
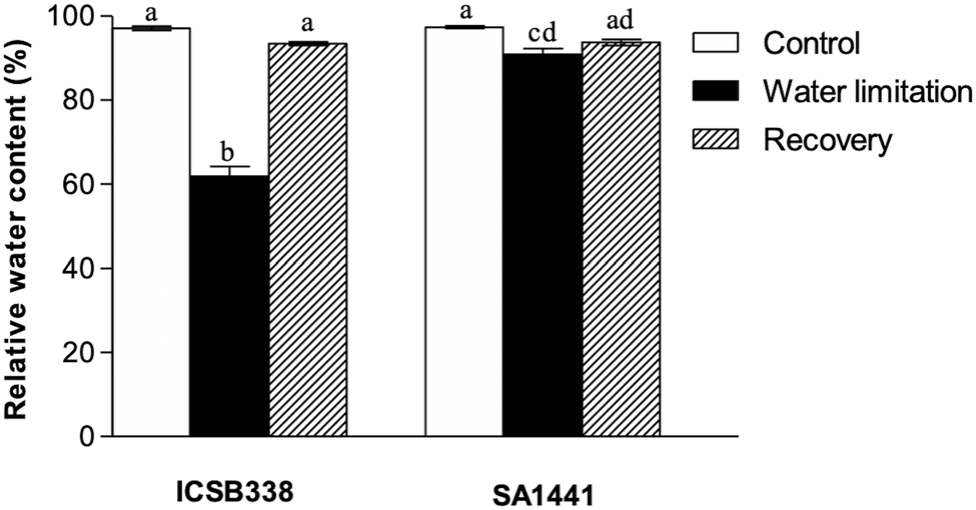
Effects of water limitation on leaf RWC of sorghum. The RWC for the control and drought stressed leaf samples of ICSB338 (drought-susceptible) and SA1441 (drought-tolerant) sorghum varieties was measured on day 8 of water limitation. The seedlings were re-watered and the measurements were repeated after 24 h. Bars represent mean ± SE (*n* = 5). Bars with the same letter are not significantly different at *p* ≤ 0.05 according to the Mann–Whitney test.

We observed a statistically significant reduction in the leaf relative water content (RWC) of both sorghum varieties eight days after water was withheld (Fig. 2). However, there was a greater reduction in RWC of the drought-sensitive variety ICSB338 when compared to that of the drought-tolerant SA1441 variety. The water deficit was fully alleviated by re-watering, with full hydration achieved in previously water-limited plants of both varieties 24 h after re-watering (Fig. 2). This shows that the water limitation imposed under these experimental conditions did not inflict permanent damage to the roots and root function.

**Figure 3:**
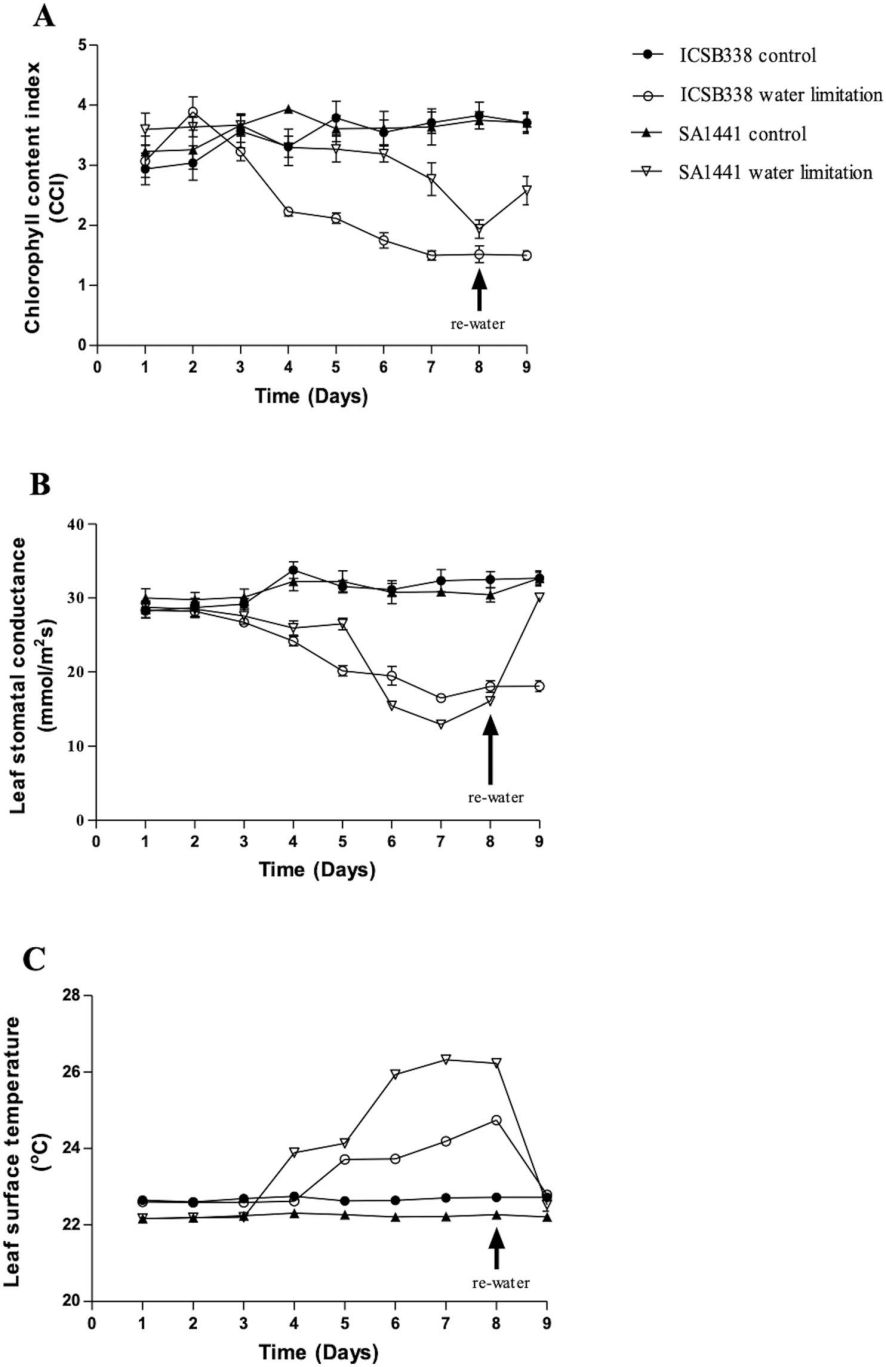
Effects of water limitation on physiological parameters of sorghum. The leaf chlorophyll content (**A**), leaf stomatal conductance (**B**), and leaf surface temperature (**C**) were measured from day 1–8 of water limitation and 24 h after re-watering. ICSB338 is drought-susceptible and SA1441 is drought-tolerant. Bars represent mean ± SE (*n* = 5).

Differences in the leaf chlorophyll content, stomatal conductance and leaf surface temperatures were also observed between the two sorghum varieties during water limitation as shown in Fig. 3A–C, respectively. Three days after withholding water, there was no significant difference in the chlorophyll content indices of the water-stressed plants of both sorghum varieties relative to their controls (Fig. 3A). However, after 4 days of water-limitation, there was a sharp drop in the chlorophyll content of the drought-susceptible variety ICSB338, which continued to fall until it levelled-off at 7 days (Fig. 3A). In contrast, the drought-tolerant SA1441 maintained near-normal chlorophyll content well after chlorophyll damage had been recorded in ICSB338. The chlorophyll content of SA1441 started to decline only after 6 days and exhibited significant recovery 24 h after re-watering (Fig. 3A). In contrast, variety ICSB338 did not recover one day after re-watering (Fig. 3A).

Similar trends in the decline of stomatal conductance were observed in both sorghum varieties from the third day of water limitation (Fig. 3B). However, SA1441 exhibited tighter stomatal control compared to ICSB338 by showing full recovery after re-watering. Stomatal conductance of ICSB338 remained unchanged 24 h after re-watering (Fig. 3B), suggesting that restoration of gas exchange immediately after cessation of drought conditions may not occur. Significantly different but constant leaf surface temperatures were observed in the well-watered control plants of both varieties throughout the experiment, with SA1441 having a lower temperature profile (Fig. 3C). Once water was withheld, the plant leaf surface temperatures increased, starting after the 3rd and 4th day for SA1441 and ICSB338, respectively (Fig. 3C). The temperature rise was quicker and significantly higher in the drought-tolerant SA1441, indicating a much swifter and tighter stomatal control in this variety when compared to the drought-sensitive ICSB338 (Fig. 3C). Again, re-watering restored the leaf surface temperatures, indicating that stomatal function had not been permanently damaged by water limitation.

**Figure 4:**
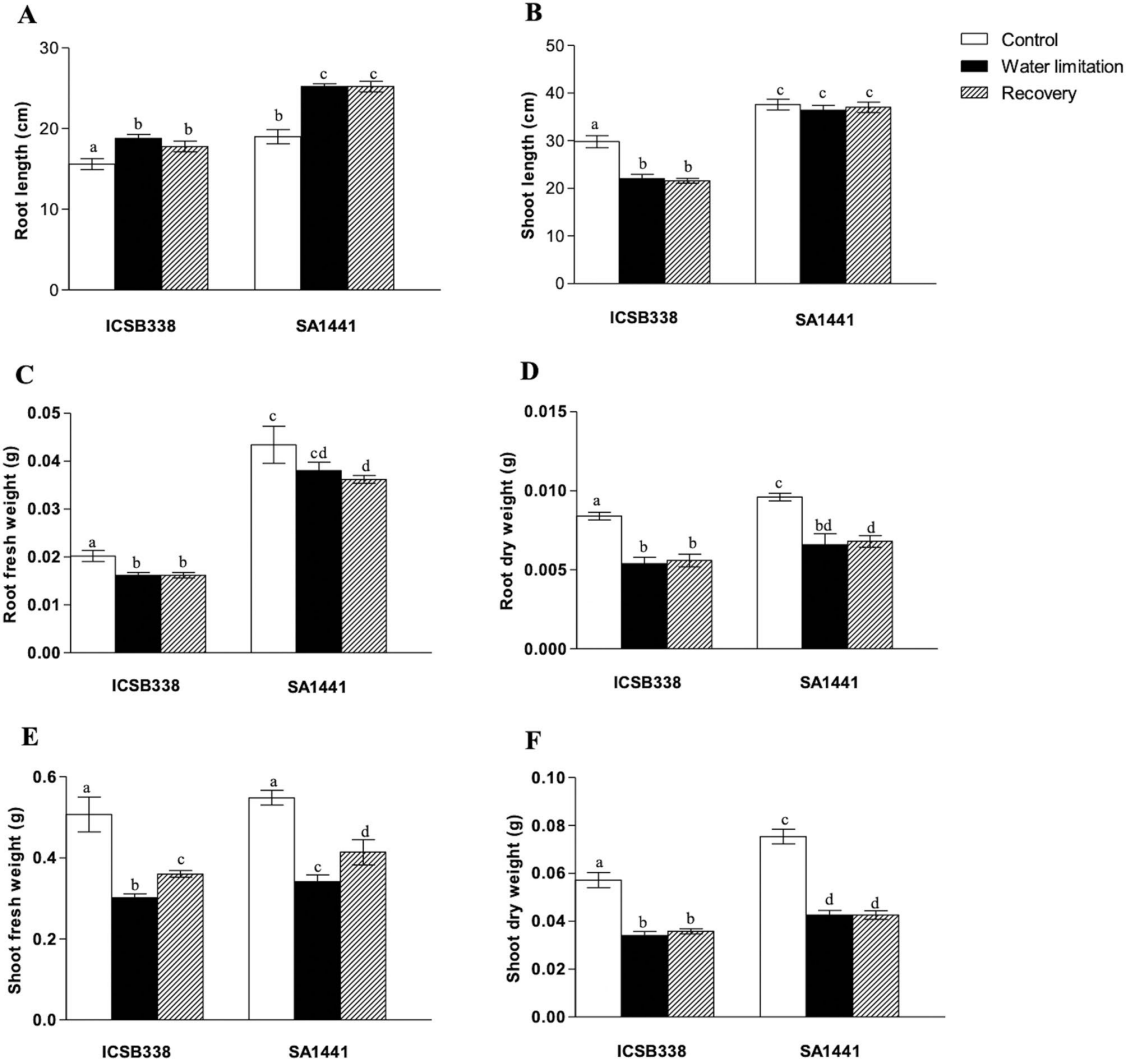
Effect of water limitation on sorghum plant growth. Root (**A**) and shoot (**B**) length, root fresh (**C**) and dry (**D**) weight, and shoot fresh (**E**) and dry (**F**) weight measurements were taken on the water-limited and re-watered plants. ICSB338 is drought-susceptible and SA1441 is drought-tolerant. Bars represent mean ± SE (*n* = 5). Bars with the same letter are not significantly different at *p* ≤ 0.05 according to the Mann–Whitney test.

We further evaluated the effects of water stress on plant growth by measuring the root and shoot length/biomass (Fig. 4). Generally, root length increased during water stress and 24 h after re-watering in both sorghum varieties (Fig. 4A). In contrast, the shoot length decreased in the water-limited plants of ICSB338 and remained unchanged after 24 h of re-watering (Fig. 4B). For the drought-tolerant variety SA1441, no significant difference in shoot length was observed between the well-watered control, water-limited and re-watered samples (Fig. 4B). With respect to biomass measurements, both sorghum varieties showed significantly reduced root and shoot fresh/dry weight for both the water-limited and re-watered samples compared to the well-watered controls (Fig. 4C–F). The only exception was SA1441 root fresh weight, which showed no significant change after water limitation (Fig. 4C). There was no significant difference in the percentage reduction of root and shoot fresh/dry weight between the two sorghum varieties following water limitation. Root fresh weight declined by ~ 18% in both sorghum varieties, while the dry weight was reduced by 35.8% and 33.1% in ICSB338 and SA1441, respectively. In contrast, shoot fresh weight declined by 39.3% and 26.8% in ICSB338 and SA1441, respectively, while the dry weight reduction estimates were 40% and 36.7% for ICSB338 and SA1441, respectively.

### Water limitation-induced osmolyte accumulation in sorghum root and leaf tissue

**Figure 5:**
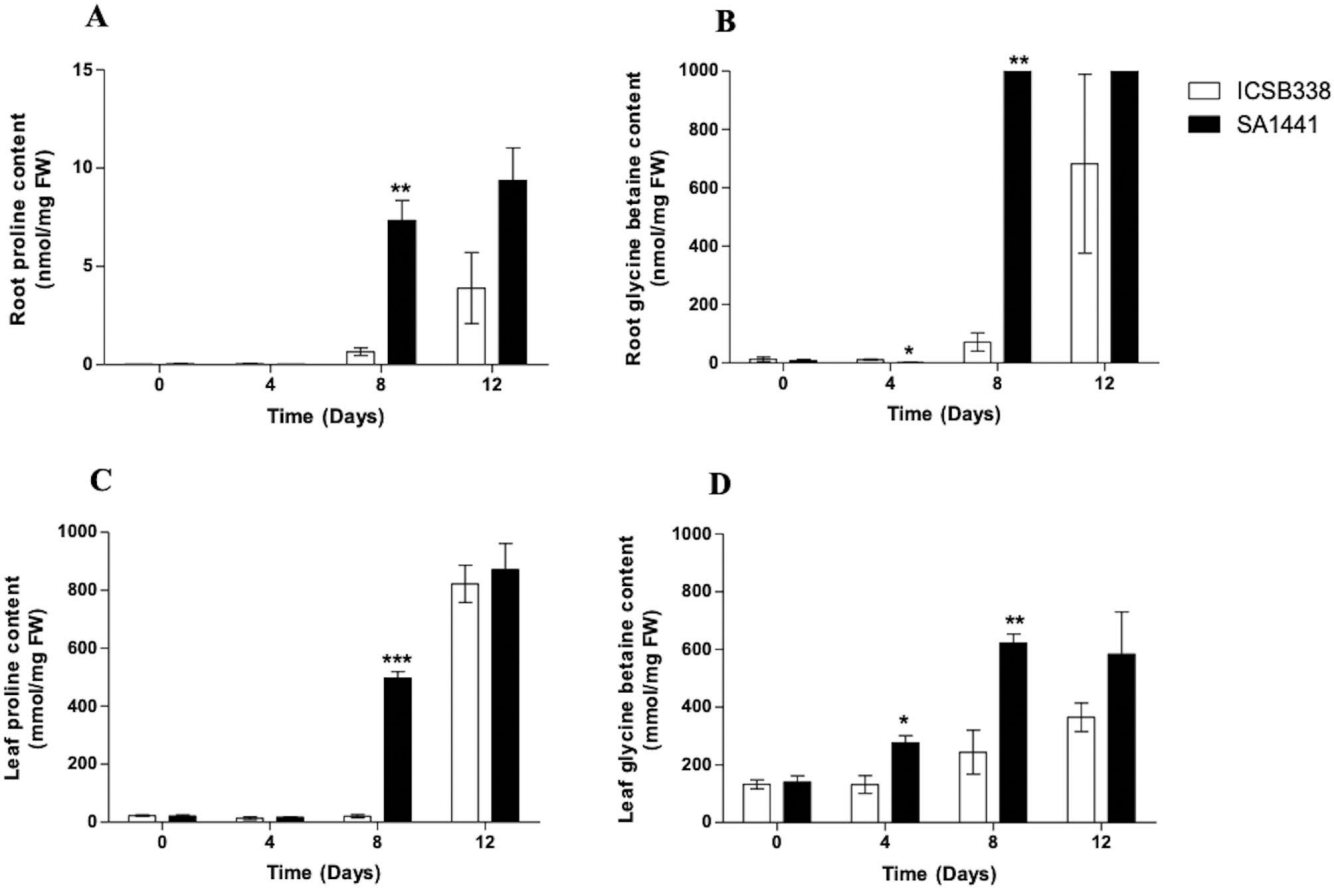
Water limitation-induced proline and glycine betaine content in sorghum. Sorghum root proline (**A**), root glycine betaine (**B**), leaf proline (**C**) and leaf glycine betaine (**D**) content for ICSB338 (drought-susceptible) and SA1441 (drought-tolerant) sorghum varieties are shown. Samples were collected at 0, 4, 8 and 12 days of water limitation for osmolyte content analysis using HILIC-MS. Bars represent ± SE (*n* = 3). *, ** and *** = a significant difference between ICSB338 and SA1441 at each time point at *p* ≤ 0.05, 0.01 and 0,001, respectively.

Next, we investigated the effects of water limitation on the accumulation of the compatible solutes proline and glycine betaine. Leaf and root samples for analysis of osmolyte content were harvested in a time-course experiment at 0, 4, 8 and 12 days after withholding water. There was a general increase of proline and glycine betaine levels in the roots and leaves of both sorghum varieties after water was withheld (Fig. 5A–D). Roots had more increased synthesis of glycine betaine than proline, and leaves had higher proline biosynthesis than glycine betaine. However, significantly higher levels of both osmolytes accumulated in the drought-tolerant variety SA1441 compared to the drought-sensitive variety ICSB338, eight days after withholding water. Again, SA1441 showed a rapid increase in osmolyte accumulation, which peaked at day 8, while the equivalent response in ICSB338 peaked at 12 days (Fig. 5A–D). Notably, more glycine betaine accumulated in SA1441 roots compared to leaves on day 8 of water limitation (Fig. 5B,D). Conversely, SA1441 leaves accumulated more proline on day 8 in comparison to roots (Fig. 5A,C). While tissue specific accumulation of proline and glycine betaine were observed, the drought-tolerant variety SA1441 had an enhanced capacity to rapidly accumulate higher levels of both compatible solutes for osmotic adjustment and/or protection of cellular components against osmotic/oxidative stress damage.

### Water limitation induces differential protein expression in sorghum root tissue

We used the isobaric tags for relative and absolute quantitation (iTRAQ) method to analyse the differentially expressed water limitation responsive root proteins. The total soluble root protein was extracted, digested with trypsin, and peptides were labelled with iTRAQ tags and analysed using liquid chromatography-tandem mass spectrometry (LC–MS/MS). Only proteins that were identified on the basis of at least two sequenced peptides were considered as positive identities. This resulted in 1,169 and 1,043 positively identified root proteins of ICSB338 and SA1441 sorghum varieties, respectively (Tables S1, S2). Database entries for over 70% of the proteins appeared to suggest that they are yet to be experimentally characterised, though we acknowledge that there are inherent delays between database updates and primary research publications that may hold relevant information. For the identification of differentially expressed root proteins, the iTRAQ datasets were analysed using the Student’s *t*-test at *p* ≤ 0.05, comparing the fold changes of the water-limited samples relative to the well-watered controls. This gave rise to 237 and 187 differentially expressed root proteins in ICSB338 (Table S3) and SA1441 (Table S4) sorghum varieties, respectively. Of these differentially expressed proteins, 111 (47%) were significantly (*p* ≤ 0.05) up-regulated whilst 126 (53%) were significantly down-regulated in ICSB338. For SA1441, 113 (61%) proteins were up-regulated and 74 (39%) were down-regulated. Tables 1 and 2 show proteins selected on the basis of a 1.5-fold change threshold that are common to both varieties, and unique to either ICSB338 or SA1441, respectively. However, the entire list of positively identified water limitation sorghum root responsive proteins of this study with a significant *p* value is in Supplementary Tables S5–S7.

**Table 1:** List of water limitation responsive sorghum root proteins common to both sorghum varieties.

Accession^a^	Protein name	ICSB338			SA1441			ICSB338 vs SA1441	Protein family^f^
		Ratio^b^	SD^c^	*p-*value^d^	Ratio^b^	SD^c^	*p-*value^d^	ratio *p-*value^e^	
**Defence/detoxification**									
C5Z469	Peroxidase OS = *Sorghum bicolor* GN = SORBI_3010G161600	1.66	0.28	4.2E−03	1.29	0.15	3.09E−02	5.64E−02	Plant peroxidase
C5XHF1	Uncharacterized protein OS = *Sorghum bicolor* GN = SORBI_3003G136200	1.65	0.15	2.7E−04	1.53	0.14	2.66E−04	2.71E−01	Germin
C5YBH7	Uncharacterized protein OS = *Sorghum bicolor* GN = SORBI_3006G135500	1.56	0.19	3.1E−03	1.57	0.23	2.75E−03	9.41E−01	Galactose oxidase, central domain family
C5YD83	Uncharacterized protein OS = *Sorghum bicolor* GN = SORBI_3006G031200	1.29	0.17	2.0E−02	1.57	0.09	3.80E−05	2,73E−02*	Thioredoxin-like superfamily
A0A1B6P7A4	Uncharacterized protein OS = *Sorghum bicolor* GN = SORBI_3009G070800	1.55	0.34	1.8E−02	1.37	0.10	6.51E−03	3.57E−01	Acid phosphatase, plant family
C5Y4L1	Pyruvate kinase OS = *Sorghum bicolor* GN = SORBI_3005G034400	− 1.51	0.06	2.9E−03	− 1.35	0.10	7.10E−03	2.28E−01	Pyruvate kinase
C9DHL2	Lipoxygenase OS = *Sorghum bicolor*	− 1.53	0.05	9.0E−06	− 1.35	0.04	7.90E−03	3.23E−02*	Lipoxygenase
C5WNH9	Uncharacterized protein OS = *Sorghum bicolor* GN = SORBI_3001G405500	− 1.71	0.04	3.2E−04	− 1.29	0.09	1.07E−02	9.47E−03*	Thiamine pyrophosphate (TPP)-dependent enzyme family
**Proteolysis**									
C5XDR4	Uncharacterized protein OS = *Sorghum bicolor* GN = SORBI_3002G217200	1.69	0.51	3.7E−02	1.47	0.17	3.24E−03	4.48E−01	Peptidase C1A family
**Transporters/intracellular traffic**									
C5XFB9	Uncharacterized protein OS = *Sorghum bicolor* GN = SORBI_3003G244200	1.52	0.30	1.6E−02	1.29	0.10	1.81E−02	2.03E−01	V-type ATPase subunit E
**Unclassified**									
C5Y1X4	Uncharacterized protein OS = *Sorghum bicolor* GN = SORBI_3005G103300	− 2.60	0.00	2.8E−04	− 1.52	0.07	1.01E−02	3.43E−04*	Zinc-containing alcohol dehydrogenase family

These proteins have a fold-change of at least 1.5 in at least one of the sorghum varieties.

^a^Protein accession numbers obtained from the UniProt database searches against sequences of *S. bicolor* only.

^b^Ratio represents the average fold-change (*n* = 4) in response to water limitation relative to the well-watered control. A negative value indicates down-regulation.

^c^Standard deviation of the fold-changes (*n* = 4).

^d^Probability value obtained from a Student’s *t*-test comparing the fold changes between the water limitation treatments and the well-watered control (*n* = 4).

^e^Probability value obtained from a Student’s *t*-test comparing the fold changes between ICSB338 and SA1441 biological replicates (*n* = 4). The four proteins marked with an asterisk (*) had significantly different fold changes between the two sorghum varieties.

^f^Family name as predicted using the InterPro (https://www.ebi.ac.uk/interpro/) and Superfamily (www.supfam.org) databases. In cases where protein families are not predicted, functional domains are listed instead.

**Table 2:** List of water limitation responsive sorghum root proteins unique to either ICSB338 or SA1441 with a fold-change of at least 1.5.

Accession^a^	Protein name	Ratio^b^	SD^c^	*p* value^d^	Protein family^e^
**Protein changes unique to ICSB338**					
**Defence/detoxification**					
C5WZ08	Uncharacterized protein OS = *Sorghum bicolor* GN = SORBI_3001G514200	1.77	0.35	1.0E−02	Thioredoxin-like superfamily
C5YGF5	Peroxidase OS = *Sorghum bicolor* GN = SORBI_3006G079300	− 1.51	0.06	8.8E−04	Plant peroxidase
A0A1B6QQQ9	Uncharacterized protein OS = *Sorghum bicolor* GN = SORBI_3001G517700	− 1.61	0.16	2.4E−02	Catalase, mono-functional, haem-containing
**Metabolism**					
C5Z6W2	Uncharacterized protein OS = *Sorghum bicolor* GN = SORBI_3010G086800	1.98	0.36	1.8E−03	Phytocyanin
Q94EL4	Chitinase-B (Fragment) OS = Sorghum arundinaceum	1.55	0.24	6.9E−03	Glycoside hydrolase, family 19
C5WRM3	Uncharacterized protein OS = *Sorghum bicolor* GN = SORBI_3001G443100	− 1.58	0.09	3.2E−03	Aldo/keto reductase
C5XX52	Glyceraldehyde-3-phosphate dehydrogenase OS = *Sorghum bicolor* GN = SORBI_3004G205100	− 1.59	0.01	3.3E−04	Glyceraldehyde 3-phosphate dehydrogenase, type 1
B5B9V8	Glutamine synthetase OS = *Sorghum bicolor* GN = gs	− 1.60	0.06	3.4E−04	Glutamine synthetase, N-terminal domain superfamily
C5XFH6	Fructose-bisphosphate aldolase OS = *Sorghum bicolor* GN = SORBI_3003G393900	− 1.64	0.03	4.9E−04	Fructose-bisphosphate aldolase, class-I
H2ET77	Sucrose synthase OS = *Sorghum bicolor* GN = SUSY2	− 1.69	0.04	2.7E−05	sucrose synthase, plant/cyanobacteria
C5YYK2	Uncharacterized protein OS = *Sorghum bicolor* GN = SORBI_3009G014600	− 1.71	0.22	3.1E−02	Phospholipase A1-II
C5YUA1	Phosphotransferase OS = *Sorghum bicolor* GN = SORBI_3009G069800	− 1.75	0.18	1.1E−02	Hexokinase
C5Y5U9	Uncharacterized protein OS = *Sorghum bicolor* GN = SORBI_3005G177500	− 1.97	0.01	1.9E−02	Glycoside hydrolase superfamily
C5Y1P4	Uncharacterized protein OS = *Sorghum bicolor* GN = SORBI_3005G099000	− 2.10	0.06	2.1E−02	Glycoside hydrolase superfamily
**Protein synthesis**					
A0A194YL15	Uncharacterized protein OS = *Sorghum bicolor* GN = SORBI_3010G230700	− 1.57	0.27	4.9E−02	Ribosomal protein L27e
C5XDZ7	Uncharacterized protein OS = *Sorghum bicolor* GN = SORBI_3002G372400	− 1.66	0.06	1.4E−03	Ribosomal protein L15
**Proteolysis**					
C5YPX8	Uncharacterized protein OS = *Sorghum bicolor* GN = SORBI_3008G134700	2.93	0.54	4.3E−04	Aspartic peptidase A1 family
C5Z8D3	Uncharacterized protein OS = *Sorghum bicolor* GN = SORBI_3010G239400	− 2.06	0.04	6.5E−04	Peptidase C1A
**Cell structure**					
A0A1B6Q2X9	Uncharacterized protein OS = *Sorghum bicolor* GN = SORBI_3003G135400	1.87	0.39	5.6E−03	Tubulin
**Unclassified**					
A0A1B6PCX7	Uncharacterized protein OS = *Sorghum bicolor* GN = SORBI_3008G106700	1.66	0.44	1.0E−02	NAD(P)-binding domain superfamily
C5XNE6	Uncharacterized protein OS = *Sorghum bicolor* GN = SORBI_3003G050200	1.57	0.38	4.2E−02	Embryo-specific ATS3
C5YXZ2	Uncharacterized protein OS = *Sorghum bicolor* GN = SORBI_3009G002400	1.53	0.31	1.6E−02	Leucine-rich repeat domain superfamily
C5YI64	Uncharacterized protein OS = *Sorghum bicolor* GN = SORBI_3007G198000	1.52	0.08	2.7E−04	Not predicted
O82430	Putative alcohol dehydrogenase 1 OS = *Sorghum bicolor* GN = Adh1	− 1.64	0.03	3.4E−03	NAD(P)-binding domain superfamily
A0A1B6QMW0	Uncharacterized protein OS = *Sorghum bicolor* GN = SORBI_3001G354200	− 2.02	0.07	1.2E−02	O-methyltransferase COMT-type family
A0A1B6PRF2	Uncharacterized protein OS = *Sorghum bicolor* GN = SORBI_3005G103500	− 3.62	0.04	5.2E−03	NAD(P)-binding domain superfamily
**Protein changes unique to SA1441**					
**Defence/detoxification**					
J7FJG8	Late embryogenesis abundant protein 3 OS = *Sorghum bicolor* GN = Lea3	2.15	0.92	4.85E−02	Late embryogenesis abundant protein family
**Metabolism**					
A0A1B6QHU0	Uncharacterized protein OS = *Sorghum bicolor* GN = SORBI_3001G073900	2.01	0.36	3.48E−03	Malate dehydrogenase, type 1
C5WN52	Uncharacterized protein OS = *Sorghum bicolor* GN = SORBI_3001G119100	1.68	0.42	2.21E−02	Phytocyanin
C5Z8E7	Uncharacterized protein OS = *Sorghum bicolor* GN = SORBI_3010G110800	1.58	0.31	1.17E−02	Phytocyanin
A0A1B6P878	Uncharacterized protein OS = *Sorghum bicolor* GN = SORBI_3009G119200	1.51	0.07	5.95E−03	Glycoside hydrolase family 17
**Transcription**					
C5WPC4	Histone H2A OS = *Sorghum bicolor* GN = SORBI_3001G416900	2.23	0.75	1.98E−02	Histone H2A
C5YM38	Histone H2B OS = *Sorghum bicolor* GN = SORBI_3007G149600	2.13	0.46	5.03E−03	Histone H2B
C5WTL6	Histone H4 OS = *Sorghum bicolor* GN = SORBI_3001G313200	2.06	0.33	7.98E−04	Histone H4
C5XX54	Histone H2A OS = *Sorghum bicolor* GN = SORBI_3004G205300	1.73	0.27	4.03E−03	Histone H2A
C5XAT9	Histone H2A OS = *Sorghum bicolor* GN = SORBI_3002G330000	1.58	0.24	7.85E−03	Histone H2A
**Protein synthesis**					
C5XMD2	Uncharacterized protein OS = *Sorghum bicolor* GN = SORBI_3003G322400	1.82	0.57	3.17E−02	Ribosomal protein S25
C5XAE4	Uncharacterized protein OS = *Sorghum bicolor* GN = SORBI_3002G049300	1.79	0.39	1.23E−02	Ribosomal protein S13
C5WW48	Uncharacterized protein OS = *Sorghum bicolor* GN = SORBI_3001G046300	1.53	0.07	5.48E−04	Ribosomal protein S5
**Proteolysis**					
A0A1B6P5R2	Uncharacterized protein OS = *Sorghum bicolor* GN = SORBI_3009G009600	1.68	0.30	4.26E−03	Proteinase inhibitor, potato inhibitor I
**Signal transduction**					
A0A1B6Q0N0	Uncharacterized protein OS = *Sorghum bicolor* GN = SORBI_3003G005800	1.66	0.18	4.54E−03	DREPP family
C5X044	Uncharacterized protein OS = *Sorghum bicolor* GN = SORBI_3001G080700	1.57	0.26	2.47E−02	Protein kinase-like domain superfamily
**Transporters/intercellular traffic**					
C5XQM5	Uncharacterized protein OS = *Sorghum bicolor* GN = SORBI_3003G367400	2.32	1.19	5.15E−01	Longin-like domain superfamily
**Unclassified**					
C5XFL0	Uncharacterized protein OS = *Sorghum bicolor* GN = SORBI_3003G397500	2.00	0.48	8.56E−03	Not predicted
C5YCX2	Uncharacterized protein OS = *Sorghum bicolor* GN = SORBI_3006G159300	1.74	0.23	1.23E−03	Not predicted
A0A1B6PJ81	Uncharacterized protein OS = *Sorghum bicolor* GN = SORBI_3007G225300	1.73	0.33	1.06E−02	Nitronate monooxygenase

^a^Protein accession numbers obtained from the UniProt database searches against sequences of *S. bicolor* only.

^b^Ratio represents the average fold-change (*n* = 4) in response to water limitation relative to the well-watered control. A negative value indicates down-regulation.

^c^Standard deviation of the fold-changes (*n* = 4).

^d^Probability value obtained from a Student’s *t*-test comparing the fold changes between the water limitation treatments and the well-watered control (*n* = 4).

^e^Family name as predicted using the InterPro (https://www.ebi.ac.uk/interpro/) and Superfamily (www.supfam.org) databases. In cases where protein families are not predicted, functional domains are listed instead.

**Figure 6:**
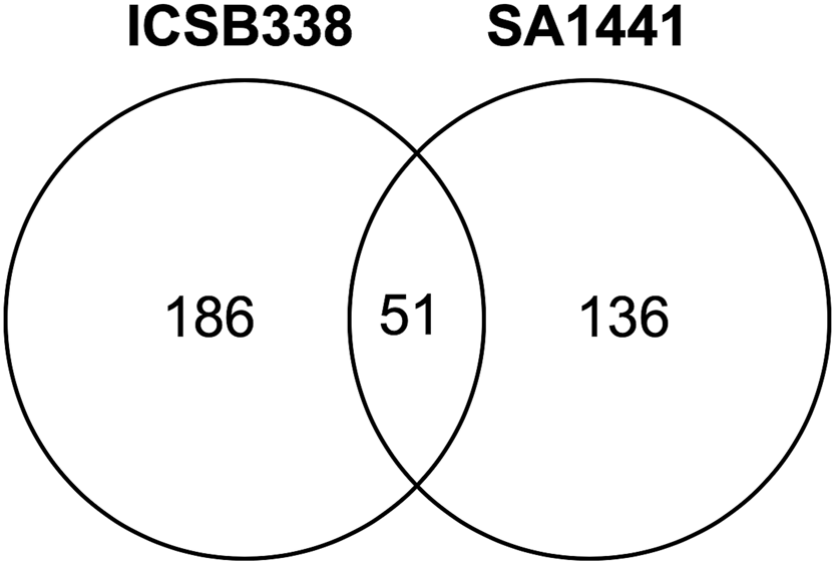
A Venn diagram of water limitation responsive sorghum root proteins.

The total number of differentially expressed proteins when the two datasets were combined is 373 proteins, with 51 of these constituting the overlap (Fig. 6). The 51 differentially expressed proteins in both ICSB338 and SA1441 are shown in Table S5. A Student’s *t*-test analysis of the average fold change of each of the 51 common proteins indicated that nine proteins (marked with an asterisk in Tables 1, S5) significantly (*p* ≤ 0.05) differed in the magnitude of change between the two sorghum varieties. We also observed two sets of protein isoforms expressed by two genes in ICSB338 and SA1441 root tissues (Table S5). The genes *SORBI_3004G166700* and *PFK* code for a glycoside hydrolase and an ATP-dependant 6-phosphofructokinase, respectively. The rest of the identified proteins responsive to water limitation were unique to either ICSB338 (186 proteins, Table S6) or SA1441 (136 proteins, Table S7) in terms of both protein accession numbers and gene identities.

We subsequently performed a computational analysis of the putative domains of all the water limitation responsive proteins in order to assign protein families for functional characterisation (Tables S5–S7). The proteins were assigned to theoretical functional groups such as defence/detoxification, metabolism, transcription, protein synthesis, proteolysis, signal transduction, transporters/intracellular traffic, cell structure, while some were unclassified. Based on the list of common water limitation responsive proteins (Table S5), two prominent expression trends were observed. The sorghum varieties responded to the water limitation by up-regulating putative signal transduction and defence/detoxification-related proteins while down-regulating those involved in metabolism. For example, out of the 17 common defence/detoxification-related proteins identified (Table S5), 13 were up-regulated in response to water limitation in both varieties, and 16 proteins were down-regulated out of the 20 common metabolism-related proteins. Two common proteins were assigned to the signal transduction functional category and both were up-regulated (Table S5).

An analysis of the unique water limitation responsive proteins of the two sorghum varieties yielded other contrasts in expression patterns of the theoretically classified proteins. Firstly, the drought-tolerant SA1441 had 11 putative transcription-related proteins, which were all up-regulated (Table S7), while none were classified in this category for the drought-sensitive ICSB338 (Table S6). Secondly, of the 26 putative defence/detoxification-related proteins identified in SA1441, 20 were up-regulated (Table S7). In ICSB338, 29 proteins were theoretically classified in the same group with only 13 being up-regulated. Thirdly, ICSB338 had 17 putative proteolysis-related proteins compared to 5 in SA1441. Of these, 12 peptidases were up-regulated in ICSB338. In contrast, SA1441 only had one up-regulated aspartic peptidase and two proteinase inhibitors, while two other peptidases were down- regulated. Fourthly, ICSB338 and SA1441 has 67 and 31 proteins that were theoretically involved in a range of metabolic processes. Of these, 44 and 19 were down-regulated in ICSB338 and SA1441, respectively. Lastly water limitation triggered the up-regulation of 20 putative protein synthesis-related proteins in SA1441 compared to 3 in ICSB338. Taken together, these proteomic results illustrate a wide variety of water limitation responsive cellular processes that are activated and/or de-activated in the two sorghum genotypes.

### Water limitation-induced root and leaf gene expression analysis

**Figure 7:**
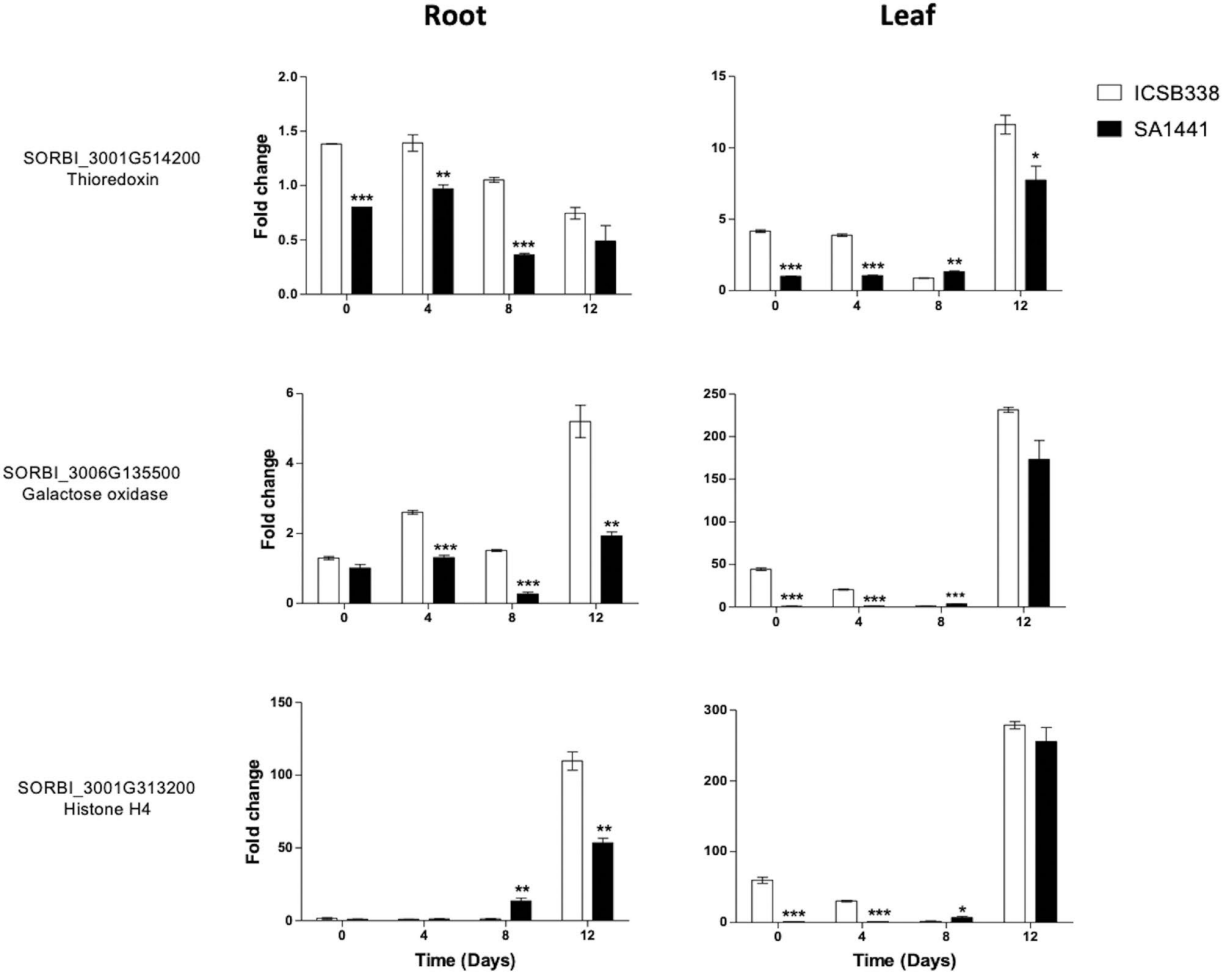
Gene expression in sorghum root and leaf tissue following water limitation. ICSB338 and SA1441 sorghum plants were exposed to water limitation by withholding water for 12 days. Root and leaf tissue samples were harvested at the indicated time points and gene expression analysis was performed using qRT-PCR. Bars represent mean ± SE (*n* = 3). *, ** and *** = a significant difference at *p* ≤ 0.05, 0.01 and 0,001, respectively.

We then performed gene expression analysis in water-limited sorghum plants in order to validate the water stress-induced protein changes observed in the roots (Tables S5–S7). A time-course water limitation experiment was conducted by withholding water for 12 days. Root and leaf samples were harvested at 0, 4, 8 and 12 days after water was withheld for gene expression analysis. We selected three genes *SORBI_3006G135500, SORBI_3001G514200* and *SORBI_3001G313200* from the water limitation responsive protein lists (Table S5-S7) for quantitative real-time PCR (qRT-PCR) analysis. These targets were selected on the basis of high fold-changes observed for their respective proteins in the iTRAQ experiment and also high specificity of their primer pairs. *SORBI_3006G135500 (galactose oxidase)* was commonly found in both sorghum varieties (Table S5), while *SORBI_3001G514200* (*thioredoxin)* (Table S6) and a *SORBI_3001G313200 (Histone H4)* (Table S7*)* were unique to ICSB338 and SA1441, respectively. The gene expression results are shown in Fig. 7. Overall, all three genes showed differential expression between ICSB338 and SA1441 in at least two time points in both leaf and root tissues. The response of these genes to water limitation was significantly lower in the drought-tolerant SA1441 than the sensitive ICSB338. A noteworthy observation is that the end-point expression at 12 days shows a remarkable difference between the two tissue types. Water limitation-induced gene expression was significantly lower in SA1441 roots for genes *SORBI_3001G313200* and *SORBI_3006G135500* 12 days after water was withheld*.* However*,* in leaf tissue, these genes were expressed at similar levels to that of ICSB338 at day 12 (Fig. 7).

## Discussion

Two sorghum varieties were chosen for their differential responses to drought from sorghum breeding screens. Although cereal crop breeding focuses on developing more drought-tolerant cultivars, identifying sorghum lines provides a vital resource for intra-species comparative studies to understand the crucial adaptive responses sorghum deploys for drought survival. We used a range of carefully selected physiological and biochemical parameters to gain better insight into the coordination of drought responses between sorghum roots and leaves.

Stomata play a critical role of balancing water conservation and enabling photosynthesis and respiration via regulating gaseous exchange. When water-limitation was imposed on the plants during this study, the drought-tolerant SA1441 retained significantly higher levels of water in leaves than the drought-sensitive ICSB338. Cellular hydration was estimated using the leaf RWC, a parameter that measures the plant water status^40^. The observed high RWC in SA1441 (Fig. 2) was partly attributable to stomatal control of water loss (Fig. 3B). The drought-sensitive ICSB338 was less effective than the drought-tolerant SA1441 in controlling stomatal responses as indicated by the prolonged delay in the reduction of stomatal conductance or the rise in leaf surface temperature, parameters which reflect stomatal closure/opening^2,6,8,41^. Since stomatal control is closely associated with water use efficiency by balancing water lost during transpiration and carbon absorbed for photosynthesis^42^, the observed low stomatal conductance (Fig. 3B) and high RWC (Fig. 2) in SA1441 highlights the superior drought phenotype of the variety compared to ICSB338. Similar results were previously reported in drought-tolerant sorghum^35^ and rice^43^.

When soil water starts to diminish, the osmotic potential across the root cell plasma membrane can reverse and stop water absorption by the roots. One strategy to maintain water uptake in progressively drying soil is synthesis and accumulation of organic solutes, such as proline and glycine betaine, for osmoregulation as well as protection of macromolecules against drought-induced osmotic and oxidative damage^9,13,14,44,45^. While the amino acid proline widely occurs in plants^44^, many plants such as rice, tobacco, potato and Arabidopsis are non-accumulators of glycine betaine^13,46^. However, glycine betaine accumulates in sorghum under control conditions or salt stress^47^. A positive correlation between high proline content and the stay-green trait has been identified in sorghum^48^. The stay-green trait is also associated with drought tolerance as plant leaves retain chlorophyll and photosynthetic activity after anthesis^22,49,50^ under water deficits. In maize, the role of glycine betaine in drought stress response was investigated using transgenic and wild-type lines. The transgenic maize line was transformed with a *betA* gene, which encodes for choline dehydrogenase, a key enzyme in the biosynthetic pathway of glycine betaine. The results showed that transgenic maize plants accumulated higher levels of glycine betaine and were more drought-tolerant compared to non-transgenic wild-type maize plants^51^. Because of the protective and osmoregulatory functions of both proline and glycine betaine in stress response^9,13,14,44,45^, their significantly higher accumulation in SA1441 compared to ICSB338 on day 8 after initiation of water limitation (Fig. 5) could contribute towards the maintenance of optimal water status, cell structure and function, and ultimately plant growth^8^. Similar trends in higher accumulation of proline in drought-tolerant sorghum varieties have been previously reported in a drought stress experiment^34^.

Sorghum is known for maintaining its yield output even under drought conditions, and this requires protection of the photosynthetic machinery from drought-induced osmotic and oxidative damage known to disrupt photosynthetic pigments^6,8^. Chlorophyll appeared to be well-protected in the drought-tolerant SA1441 variety, which could be attributable to both retention of leaf water (Fig. 2) and rapid synthesis of the protective proline and glycine betaine osmoprotectants in leaves (Fig. 5). Drought-tolerant sorghum varieties often have the stay-green trait^22,49,50^, which is associated with accumulation of osmolytes^48^ and greater protective capacity of the photosynthetic apparatus. Rong-hua and co-workers^52^ evaluated changes in chlorophyll content of barley varieties in response to limited watering after flowering, in a soil pot experiment. Results showed that the chlorophyll content of drought-sensitive barley varieties declined by 30.1 and 31.3%, while the drought-tolerant varieties reduced chlorophyll content by modest levels of 1.6 and 10.7%^52^.

During the current study, changes in plant growth patterns were monitored by measuring the length and weight of roots and shoots (Fig. 4). The observed increase in the root/shoot ratio (Fig. 4) is an adaptive feature for enhanced soil water uptake in order to maintain favorable cellular hydration during drought stress^8,53–56^. In the current study, the shoot length only significantly decreased in the water-limited and re-watered plants of the drought-sensitive ICSB338 sorghum variety compared to the drought-tolerant SA1441 (Fig. 4B). This result possibly indicates a compromised photosynthetic capacity of the drought-sensitive ICSB338 sorghum due to a decline in chlorophyll content (Fig. 3A) and a reduction in cellular hydration levels (Fig. 2), which in turn affects cell expansion and growth^8^. However, the shoot and root biomass of both sorghum varieties decreased with water limitation (Fig. 3C–F). This observation is consistent with reports by Blum^53^ who states that root length and depth may increase in dry conditions even with a reduced total root mass. Such a physiological response could be attributed to an existing trade-off between mechanisms of drought avoidance and biomass accumulation under water deficits^54^. In sorghum, the amount of starch present in bundle sheath chloroplasts decreased with an increase in water stress^57^. The root fresh weight was twofold higher in control plants of SA1441 compared to ICSB338 but not so pronounced in dry weight (Fig. 4C,D). While the cause for this difference may not be apparent in the current study, future work on root morphology using electron microscopy is required. Taken together, the measured physiological parameters confirmed the drought-tolerant nature of SA1441 and the drought-sensitivity of ICSB338. The water limitation experiment in deeper pots also confirmed the contrasting drought phenotypes of the sorghum varieties (Fig. 1). When watering was withheld, the soil started drying from the top, simulating the receding water table under field conditions. Drought tolerant SA1441 roots can continue to grow downwards, following the receding water table and plants survive (Fig. 1D). In contrast, ICSB338 roots become sensitive to water stress and eventually die (Fig. 1C).

We performed a comparative root proteome analysis of the two sorghum varieties following 8 days of water limitation. We observed three notable trends amongst the 51-common water limitation responsive sorghum root proteins (Table S5). Sorghum plants, irrespective of their drought phenotype, responded to the imposed stress by up-regulating the expression of signal transduction, and defence/detoxification-related proteins, while down-regulating those involved in metabolic processes. As the root system senses osmotic stress, signaling cascades are transmitted across cells and tissues to activate specific physiological responses^58,59^. ABA is also an important root-to-shoot stress signal that is implicated in stomatal closure, restricted leaf growth^56,60^ and drought-induced gene expression^10–12^. In this study two un-characterised sorghum proteins belonging to a nucleoside diphosphate kinase family and a C2 domain superfamily were commonly up-regulated in both sorghum varieties (Table S5). Other signaling proteins unique to either ICSB338 (Table S6) or SA1441 (Table S7) were also up-regulated in this study. These include another C2 domain-containing protein and an adenylate-kinase/UMP-CMP kinase identified in ICSB338 (Table S6), whilst proteins belonging to the developmentally regulated plasma membrane polypeptides (DREPP), protein kinase-like domain and adenylate-kinase/UMP-CMP kinase families were identified in SA1441 (Table S7). C2 domain containing proteins, such as kinases are involved in membrane trafficking and signal transduction processes possibly via Ca^2+^ ion-dependent interactions with membranes^61,62^. Since osmotic stress triggers calcium signaling^63^, the C2 domain-containing proteins together with various other kinases identified (Tables S5-S7) may be involved in transducing signals in cells for stress responses to be elicited.

Osmotic stress induces the overproduction and accumulation of reactive oxygen species (ROS) in cells, which cause oxidative damage. At low concentrations, ROS function as signaling molecules^64^, while elevated levels cause oxidative damage to cellular macromolecules such as lipids, proteins and nucleic acids^65,66^. Plants have developed a variety of enzymatic and non-enzymatic antioxidant systems, which detoxify ROS under stress conditions^65,66^. In this study, both sorghum varieties increased the expression of peroxidases, thioredoxins, glutathione-s-transferase and germins (Table S5), which are pivotal in ROS metabolism. The up-regulation of antioxidant enzymes has also been reported in cell suspension cultures derived from white sorghum grain in response to osmotic stress^36^ as well as in sorghum root responses to aluminum stress^67^. Furthermore, a survey of the unique defence/detoxification-related proteins identified in either of the two sorghum varieties (Tables S6, S7) showed other notable differences in expression patterns. For example, the drought-tolerant SA1441 had a higher up-regulation of putative defence/detoxification-related proteins, including 10 class III peroxidases (Table S7) compared to the only two in the drought-susceptible variety ICSB338 (Table S6). In another comparative study of two sorghum lines in response to aluminum stress, the aluminum-tolerant line SC566 had an increased up-regulation of antioxidant enzymes particularly peroxidases compared to the aluminum-sensitive line BR007^67^. Type III peroxidases are involved in various physiological processes including H_2_O_2_ metabolism, cell wall lignification and suberization, auxin metabolism and in plant defence^68,69^. The rapid up-regulation of peroxidases in SA1441 could highlight amongst others, an overall enhanced capacity to balance ROS homeostasis for cellular protection against oxidative damage.

Drought stress also affects cellular metabolism. While proteins involved in carbohydrate metabolism such as glycoside hydrolases were up-regulated in both sorghum varieties, those involved in the glycolytic pathway, amino acid biosynthesis and fatty acid metabolism were down-regulated (Table S5). A regulation of these metabolic pathways could facilitate the channeling of cellular energy towards signaling pathways and the protection of cellular machinery against osmotic and oxidative stresses. Although both sorghum varieties responded to the water stress by down-regulating glucose, amino acid, and fatty acid metabolic processes, the decrease was more pronounced in the drought-susceptible variety ICSB338 (Table S6). While the role of this differential inhibition in response to water limitation may not be apparent in the current study, further studies using metabolomic profiling of the sorghum varieties may elucidate this process.

Other notable trends were identified amongst the unique proteins of ICSB338 (Table S6) and SA1441 (Table S7) in response to water limitation. Firstly, 11 transcription-related proteins were identified in the drought-tolerant variety and all were up-regulated in response to the water limitation treatment. Of these, six were histone proteins and three nascent polypeptide-associated complex (NAC) subunit beta proteins (Table S7). Histones are structural proteins involved in the packaging of DNA molecules into chromatin^70^, which stabilizes and condenses the DNA^71^. Chromatin is modified during stress response, an important process in transcriptional control of stress-related genes^71^. A wide range of stress-inducible transcriptional factors, including NAC family proteins have been associated with abiotic stress response in plants^72^. Since drought alters gene expression, the up-regulation of histone proteins and NAC transcription factors could have downstream effects on gene expression during stress response and tolerance in the drought-tolerant SA1441.

During stress response, changes in gene expression ultimately result in specific proteins being synthesized, which play important roles in stress adaptation^10^. Stress responsive proteins may include signaling proteins, proteins involved in regulating gene expression, antioxidant enzymes, chaperones and enzymes involved in the biosynthesis of organic solutes^9–12^. However, the amount of proteins present in cells at any one point largely depends on the balance between the rate of protein synthesis and the rate of protein degradation^73^. Protein degradation is largely regulated by the activities of proteases and protease inhibitors^73–76^. In this study, distinct expression patterns of protein synthesis and proteolysis-related proteins were identified in the two sorghum varieties in response to water limitation. The drought-tolerant variety SA1441 responded to the imposed water limitation treatment by up-regulating the expression of protein synthesis-related proteins, up-regulating protease inhibitors and down-regulating peptidases, thus limiting protein degradation (Table S7). Conversely, during water limitation, the drought-sensitive ICSB338 sorghum plants possibly decreased the rate of protein synthesis by down-regulating protein synthesis-related proteins and increased their degradation by up-regulating the expression of peptidases (Table S6). While proteolysis plays an important role in protein turnover^73–77^, the differential expression of proteolysis-related proteins observed in the two sorghum varieties remains unclear and requires elucidation using gene functional studies.

Analysis of gene expression patterns of three target genes, a thioredoxin, galactose oxidase and a histone indicated differential expression patterns in the sorghum varieties and between leaf and root tissues (Fig. 7). The observed tissue specific gene expression patterns possibly indicate variations in the effect of water deficits on different parts of a plant. However, larger gene expression and functional studies are required in order to evaluate the contributions of the genes in drought adaptation.

In conclusion, we used two sorghum varieties, to identify differences in physiological, biochemical and molecular mechanisms that could contribute towards sorghum responses to water limitation. Physiologically, the drought-tolerant variety SA1441 controls stomatal opening and maintains favorable plant water status. In addition, both sorghum varieties accumulated high levels of proline and glycine betaine, which may function as osmoprotectants and osmolytes. However, analysis of the root proteome revealed common and unique proteins in the two sorghum varieties in response to water limitation. As such, future work involving larger gene expression and validation studies are required in order elucidate their function in drought response. Furthermore, as the basis for the different response to water limitation seen in these sorghum varieties is still unclear, a line of investigation that may provide further insight could be focusing on ABA signaling. This would require measuring ABA levels during stress response and analysing expression of putative ABA receptors and the downstream Type 2 protein phosphatase (*PP2C*) and the sucrose *non*-fermenting 1-related protein kinase 2 (*SnRK2*) genes. Overall, the common protein expression patterns observed in this study could reflect basal stress adaptive responses in a plant species, while the unique patterns identified in contrasting sorghum varieties could possibly highlight mechanisms that either promote tolerance or susceptibility to the imposed stress.

## Methods

### Plant materials, growth conditions and water limitation treatments

Seeds of two sorghum varieties SA1441 (drought-tolerant) (race: *Kafir*, origin South Africa) and ICSB 388 (drought-susceptible) (race: *Caudatum*, from the International Crops Research Institute for the Semi-Arid Tropics (ICRISAT)-India) were obtained from the Agricultural Research Council-Grain Crops Institute, Potchefstroom, South Africa. The seeds were sown in potted soil and grown at 25–30 °C under a 16/8 h light/dark photocycle. For physiological and proteomic experiments, 10 plants per pot were grown in 400 cm^3^ plastic pots, while for gene expression and osmolyte content analysis, one plant per pot was grown in 216 cm^3^ pots. The physiological and proteomic pot experiments used a South African potting soil mix (Culterra, Muldershift, South Africa) while the gene expression and osmolyte pot experiments used Levington F2 + Sand Compost (ICL Ltd., Ipswich, UK). Levington F2 is a compost with traces of silver sand for better drainage and dolomitic lime for adjusting pH between 5.3—6.0. It is also pre-treated with fertiliser at rates of N-144, P-73, K-239 mg/L. The particle size is below 3 mm. The South African compost consists of a slow-release fertiliser, coco peat and organic raw materials. The pot size, soil type, and number of plants per pot were matched to ensure that wilting symptoms occurred at approximately the same time after withholding water. We also conducted a seedling experiment in 2.5 L pots of 22 cm depth in order to evaluate the effect of water limitation and top soil drying on survival rate of the sorghum varieties. Four-day old seedlings were transplanted into the top soil of the Levington F2 + Sand Compost (ICL Ltd) and well-watered for establishment. No watering was provided thereafter.

The plants were well-watered until the V3 stage (3 fully expanded leaves with the fourth one emerging) and divided into two groups for the control and water limitation treatments. Water limitation was imposed by withholding water for a maximum of 12 days, while control plants were well-watered throughout the experiment. The extent of water stress in the sorghum varieties was estimated by visually assessing the plants for chlorosis, wilting and leaf rolling, and by measuring physiological, growth and biochemical parameters. The plants were harvested at different time-points during the water limitation treatment for physiological, growth, biochemical, protein and gene expression analyses. For physiological and growth measurements, the plants had water withheld for eight days and some of these plants were subsequently re-watered for 24 h in order to assess their ability to recover from the imposed stress. The latter group was used as the re-watered plants in these measurements. For proteomic analysis, the plants were harvested after 8 days of water limitation treatment. Four biological replicates were prepared each being a pool of root tissue from at least 10 plants. For osmolytes and gene expression analyses, the plants were sampled in a time-course experiment at 0, 4, 8 and 12 days after water was withheld. Three biological replicates for the leaf and root tissues were prepared for osmolyte and gene expression analysis. For osmolyte analysis, each biological replicate was a pool of three 7 mm leaf discs, each derived from an independent plant. For the roots, a biological replicate consisted of 100 mg of ground fresh tissue generated from a single plant. For gene expression analysis, the third leaf and the intact root system of each single plant constituted a biological replicate.

### Determination of relative water content (RWC)

The leaf RWC was estimated as described previously^40^. Briefly, five biological replicates of the third oldest leaf of each sorghum variety were harvested eight days after the initiation of water limitation. The leaf samples were immediately weighed to determine the fresh weight (FW). The samples were immersed in 50 mL Falcon tubes containing distilled water and incubated under dark conditions at 4 °C overnight, blotted dry and weighed to determine the turgid weight (TW). The leaf samples were then oven-dried at 60 °C for 24 h to obtain the dry weight (DW). The RWC was estimated in percentage using the formula RWC = (FW − DW)/(TW − DW)*100.

### Determination of chlorophyll content, stomatal conductance and leaf surface temperature

The leaf chlorophyll content was measured using a CCM 200 Plus Chlorophyll Content Meter (Opti-Science, ADC BioScientific Ltd., Hoddesdon, UK) according to the manufacturer’s instructions. The leaf abaxial stomatal conductance and leaf surface temperature of the plants were simultaneously measured using an SC-1 Leaf Porometer (Decagon Devices, Inc., Washington, USA) according to the manufacturer’s instructions. All three measurements were taken daily at the same time of the day on the third oldest leaf for eight consecutive days during water limitation treatment and 24 h after re-watering. Ten biological replicates were sampled per treatment group for each sorghum variety.

### Plant growth measurements

Sorghum plants were harvested and the shoot and root systems were separated for length and weight measurements. The shoot length was measured from the base of the shoot to the tip of the growing point. The root length was measured from the base of the shoot to the tip of the longest root. The fresh shoot and root weight was measured immediately after harvest. The intact root tissues were gently shaken to remove excess soil, briefly washed with water and gently blotted dry using paper towels before determining the fresh weight. Shoot and root tissues were separately oven-dried at 60 °C for 48 h and the dry weight was measured. Five biological replicates were used per treatment for all length/weight measurements per sorghum variety.

### Estimation of osmolyte contents

For each of the control and water-limited plants, 7 mm leaf diameter discs and 100 mg of ground fresh root samples were used. Three biological replicates for the leaf and root tissues were harvested at days 0, 4, 8 and 12 after the initiation of water limitation and weighed. Thereafter, 125 µL of 0.25 N HCl was added to each sample and incubated at 60 °C on a heat block for 5 min as described previously^78^. The liquid extract was subsequently collected by centrifugation at 9,400×*g* for 5 min and analysed for osmolyte content using the Hydrophilic Interaction Liquid Chromatography-Mass Spectrometry (HILIC-MS). The chromatographic separation of the leaf and root samples for proline and glycine betaine were performed as described previously^79^ and the amino acid content was analysed on a QTRAP 6500 MS (Applied Biosystems Sciex, Foster City, USA) using Multiple Reaction Monitoring (MRM). A full description of the HILIC-MS method can be found as Supplementary Method online.

### Root protein extraction, preparation and iTRAQ labelling

Four biological replicates were prepared for the control and water-limited root samples of each sorghum variety. Total soluble root proteins were extracted from fresh root tissue (1 g) in 1 mL of a buffer (9 M urea, 2 M thiourea and 4% (w/v) CHAPS), by vortexing overnight as described previously^80^. The protein samples were labelled with iTRAQ tags as described previously^81^, with minor modifications. Briefly, 50 μg of protein from each sample were reduced with tris(2-carboxyethylphosphine) (TCEP) and alkylated with methyl-methane-thiol-sulfonate (MMTS). Thereafter, protein samples were digested overnight at 37 ºC using a 1:10 (w/w) trypsin to protein sample ratio, vacuum-dried, re-suspended in triethylammonium bicarbonate buffer (pH 8.5), and labelled with an 8-plex iTRAQ reagent kit (Sciex) according to the manufacturer’s instructions.

For each of the two sorghum varieties, peptides of the four control replicates were labelled with 113, 114, 115, and 116 iTRAQ tags, while the four water-limited replicate samples were labelled with 117, 118, 119, and 121 tags. The eight samples were separately pooled into one composite sample and vacuum-dried. The samples were cleaned using HILIC SPE cartridges (PolyLC Inc.), containing 300 mg of 12 µm polyhydroxyethyl-A, to remove unincorporated label and buffer salts. The cartridges were equilibrated by sequential addition of 4 × 3 mL releasing solution (5% ACN, 30 mM ammonium formate pH 3.0) followed by 4 × 3 mL binding solution (85% ACN, 30 mM ammonium formate pH 3.0). The dried iTRAQ-labelled peptide residue was dissolved in 75 µL of 3% acetonitrile (ACN), 0.1% formic acid (FA) followed by 150 µL of 0.3 M ammonium formate, pH 3. The pH of the mixture was adjusted to 3.0 using trifluoroacetic acid (TFA). After clarifying by centrifugation (10,000×*g*, 10 min), the samples were mixed with 1,275 µL ACN. The resulting 1.5 mL sample was added to the SPE cartridge and the flow-through retained and passed through a second time. The column was then washed twice with 2 mL binding solution. Finally, the peptides were eluted with 2 × 1 mL releasing solution. The eluate was freeze-dried and re-suspended in 3% ACN, 0.1% formic acid for liquid chromatography-mass spectrometry (LC–MS).

LC–MS analysis was performed using a TripleTOF 6600 mass spectrometer (Sciex) linked to an Eksigent 425 LC system via a Sciex Duospray source. Peptides originating from 5 µg protein were used for each LC–MS run and chromatographic separations of peptides used a trap and elute method. Samples were loaded and washed on a Triart C18 guard column 1/32″, 5 µm, 5 × 0.5 mm (YMC) acting as a trap, and online separation of peptides performed over 87 min on a TriArt C18 1/32″, 3 µm, 150 × 0.3 mm column (YMC) at a flow rate of 5 µL/min. Buffer A was 0.1% FA in water and buffer B 0.1% FA in ACN. Sequential linear gradients of 3 to 5% B over 2 min, 5 to 30% B over 66 min, 30 to 35% B over 5 min and 35 to 80% B over 2 min were followed by a 3 min column wash in 80% B. Return to 3% B was over 1 min before column re-equilibration for 8 min. Data-dependent top-30 MS–MS acquisition, with collision energy adjusted for iTRAQ-labelled peptides, was started immediately upon gradient initiation and was for 85 min. Throughout this period, precursor-ion scans (400 to 1,600 m/z) of 250 ms enabled selection of up to 30 multiply-charged ions (> 500 cps) for CID fragmentation and MS/MS spectrum acquisition (m/z 100–1,500) for 50 ms. The cycle time was 1.8 s and a rolling precursor exclusion of 15 s was applied to limit multiple fragmentation of the same peptide. Analyst TF 1.7.1 instrument control and data processing software (Sciex) was used to acquire spectrometer data.

### Mass spectra data analysis

Protein identification and relative quantification was performed by processing the raw .wiff data-files against the TrEMBL database of *S. bicolor* only sequences downloaded in October 2013 using ProteinPilot 5.0.1 version 4895 software, incorporating the Paragon Algorithm 5.0.1.0.4874, (AB Sciex). An iTRAQ 8-plex (peptide-labelled) Paragon method, for tryptic peptides with MMTS cys-modification and data acquired on a TripleTOF 6600 spectrometer, was used. Label bias-correction was activated in this, the ‘Thorough ID’ and ‘Run False Discovery Rate Analysis’ options were selected, and the Detected Protein Threshold was set at 0.05 (10%) [Unused ProtScore (conf)]. Peptide and protein tables were exported from ProteinPilot for subsequent manual data-handling and filtering. For each identified peptide, a minimum threshold of 1.3 at 95% confidence interval was set whilst a minimum score threshold of 2.0 at 99% confidence interval was set for protein identification. All proteins identified on the basis of a single peptide were removed from the dataset. For quantitative analysis of the differentially expressed water-limitation responsive proteins, the abundance of each protein in each sorghum variety was obtained as a ratio to the 113-tagged sample. Average ratios of each protein were subsequently calculated for the four biological replicates per sorghum variety. A negative sign indicated down-regulated proteins and the water-limited sample averages were the numerators with the controls as the denominators. A Student’s *t*-test at *p* ≤ 0.05 was used for calculating the probability values of the differentially expressed proteins.

### Bioinformatic analysis

The Gene Ontology, conserved domains and family names of the identified proteins were determined using data available on the Interpro^82^ database.

### Gene expression analysis

Total RNA was extracted from sorghum root and leaf tissue samples using the Spectrum Plant Total RNA Kit (Sigma-Aldrich, St Louis, USA) according to the manufacturer’s instructions. Complementary DNA (cDNA) synthesis was performed on 2 μg total RNA template using the GoScript Reverse Transcriptase System (Promega, Southampton, UK) according to the manufacturer’s instructions. The qRT-PCR was performed on eightfold dilution cDNA using the SensiFAST SYBR No-ROX Kit (BIOLINE) as described previously^36^. The reactions were performed on a Corbett Rotor-Gene 6000 (Qiagen, Cambridge, UK) using the following thermal cycling conditions: initial denaturation at 95 °C for 90 s followed by 45 cycles of denaturation at 95 °C for 10 s, annealing at 56 °C for 15 s and elongation at 72 °C for 25 s. All reactions were carried out for three biological replicates, each with three technical replicates. Data analysis was performed using the REST2009 software version 2.0.13 (Qiagen) and two sorghum genes, an EIF4a1 Sb04g003390^83^ and an uncharacterised Sb03g038910^33^ were used as the constitutive reference controls. A list summarizing the protein numbers, accession, gene identities, fold changes and protein family data for the four target genes is shown in Table S8. The gene specific primers were designed on the National Centre for Biotechnology Information (NCBI) database using the Primer-BLAST software^84^. The primers for all the target genes are shown in Table S9.

### Statistical analysis

The Mann–Whitney U test at *p* ≤ 0.05 was used to compare the means for physiological and biochemical results, using the GraphPad Prism 5.00 software. The Student’s *t*-test at *p* ≤ 0.05 was used to compare the protein and gene expression fold changes using Microsoft Excel version 15.41.

## Supplementary information

Supplementary file1 (PDF 195 kb)

Supplementary file2 (XLSX 1221 kb)

Supplementary file3 (DOCX 13 kb)

Supplementary file4 (DOCX 13 kb)

## Data Availability

The datasets generated and/or analysed during the current study is available from the corresponding authors on request.
