# Supplementary material for: Comparative physiological and root proteome analyses of two sorghum varieties responding to water limitation
Source: Sci Rep. 2020 Jul 16;10:11835. doi: 10.1038/s41598-020-68735-3 (PMC7366710; doi:10.1038/s41598-020-68735-3)
Supplement: Supplementary file 1 — Supplementary file1 (PDF 195 kb) [file 41598_2020_68735_MOESM1_ESM.pdf]

Title: Comparative physiological and root proteome analyses of two sorghum varieties responding to water limitation

Authors: Tatenda Goche, Nemera G. Shargie, Ian Cummins, Adrian P. Brown, Stephen Chivasa and Rudo Ngara

HILIC-MS method for proline and glycine betaine content analysis

Proline content analysis

The chromatographic separation of the leaf and root extracts for proline content analysis was performed on an Acquity UPLC BEH Amide column (1×100 mm, 1.7 µL particle size) (Waters, Milford, USA) as described previously[79]. Briefly, a volume of 2 µL of the leaf or root extracts was diluted by a factor of 100, injected into the column and the column temperature was maintained at 35°C. For optimal chromatographic separation, a gradient with two solvents, namely A (10 mM ammonium formate, 0.15% formic acid in 85% acetonitrile) and B (10 mM ammonium formate, 0.15% formic acid in distilled water, pH 3.0) was established at a flow rate of 200 µL/min as follows. Initially solvent A was maintained at 100% for 6 min. A gradient was then started for 0.1 min at which solvent A was decreased to 94.1% whilst solvent B was increased to 5.9%. Following this, solvent A was further decreased to 82.4% whilst solvent B was increased to 17.6% from 6.1 to 10 min. From 10 to 12 min, solvent A was set at 70.6% and solvent B at 29.4%. This was followed by the equilibration of the column for 6 min in 100% solvent A, giving a total run time of 18 min including the calibration process. The column was then coupled to a QTRAP 6500 MS (Applied Biosystems Sciex, Foster City, USA) and the amino acids were detected using Multiple Reaction Monitoring (MRM). The MRM transition was 116→70. Peaks of interest were integrated using Analyst software (Sciex) and quantified with reference to external standards.

Glycine betaine content analysis

The chromatographic separation of the leaf and root extract samples for glycine betaine content analysis was performed on an Ascentis HILIC column (2.1×150 mm, 1.7 µL particle size) (Supelco Analytical, Munich, Germany) as described previously[79]. Briefly, a volume of 2 µL of the leaf or root extracts was injected into the column and the temperature of the column was maintained at 30°C. For optimal chromatographic separation, a gradient with

two solvents, namely A (10 mM ammonium formate, 0.15% formic acid in 85% acetonitrile) and B (10 mM ammonium formate, 0.15% formic acid in distilled water, pH 3.0) was established at a flow rate of 200 µL/min as follows. Initial conditions were 100% solvent A for 6 min. A hold step was maintained for 2 min. Thereafter, solvent B was ramped to 100% at 5 min. This was followed by a hold step for 5 min and equilibration at 100% solvent A for 5 min. The column was then coupled to a QTRAP 6500 hybrid triple-quadrupole MS system (Sciex) for the analysis of glycine betaine content analysis using MRM. The MRM transitions were ES+ 118→58, 118→59.
