# Supplementary material for: Comparative physiological and root proteome analyses of two sorghum varieties responding to water limitation
Source: Sci Rep. 2020 Jul 16;10:11835. doi: 10.1038/s41598-020-68735-3 (PMC7366710; doi:10.1038/s41598-020-68735-3)
Supplement: Supplementary file 3 — Supplementary file3 (DOCX 13 kb) [file 41598_2020_68735_MOESM3_ESM.docx]

**Table S8:** Water limitation responsive sorghum root proteins selected for qRT-PCR analysis.

| **Accession** | **Gene Identity** | **Ratio** | | **Protein Family** |
| --- | --- | --- | --- | --- |
|  |  | **ICSB338** | **SA1441** |  |
| C5WZ08 | SORBI_3001G514200 | 1.77 | - | Thioredoxin |
| C5YBH7 | SORBI_3006G135500 | 1.56 | 1.57 | Galactose oxidase |
| C5WTL6 | SORBI_3001G313200 | - | 2.06 | Histone H4 |

- protein not differentially expressed in the sorghum variety.
