# Supplementary material for: Comparative physiological and root proteome analyses of two sorghum varieties responding to water limitation
Source: Sci Rep. 2020 Jul 16;10:11835. doi: 10.1038/s41598-020-68735-3 (PMC7366710; doi:10.1038/s41598-020-68735-3)
Supplement: Supplementary file 4 — Supplementary file4 (DOCX 13 kb) [file 41598_2020_68735_MOESM4_ESM.docx]

**Table S9:** List of sorghum target genes and primer sequences used in qRT-PCR analysis.

| **Target Gene Identity** | **Gene name** | **Primer Name** | **Forward Primer (5'>3')** | **Reverse Primer(5'>3')** |
| --- | --- | --- | --- | --- |
| SORBI_3001G514200 | Thioredoxin | SORBI_3001G514200 | GGAACATCCTGGCGCATTTG | AGAGCCAGACCGACACAAAC |
| SORBI_3006G135500 | Galactose oxidase | SORBI_3006G135500 | AGGGAAATGGTTGTACGGGC | GTGCTGGTGCACAAAATATATAGC |
| SORBI_3001G313200 | Histone H4 | SORBI_3001G313200 | GGATCCCTGGATCTTCTGGAG | TTTGGTGGCTCTGTGGTCAG |
| *Ref. gene | Sb03g038910 | Uncharacterised protein | TCCTGAAGCATCTTTCCCTCC | ACAGCCTGATTAGTTGGGGG |
| *Ref. gene | Sb04g003390 | Eukaryotic initiation factor-4A | GATGAGATGCTCTCCCGTGG | TGATCTCTAGGGCCTCTGGG |

*Reference control genes
